# Supplementary material for: Population variability in X-chromosome inactivation across 10 mammalian species
Source: Nat Commun. 2024 Oct 18;15:8991. doi: 10.1038/s41467-024-53449-1 (PMC11487087; doi:10.1038/s41467-024-53449-1)
Supplement: Supplementary file 1 — Supplementary Information [file 41467_2024_53449_MOESM1_ESM.pdf]

Population variability in X-chromosome inactivation across 10 mammalian species

Jonathan M. Werner[1,2], John Hover[1], Jesse Gillis[1,2,*]

[1]Stanley Institute for Cognitive Genomics, Cold Spring Harbor Laboratory, Cold Spring Harbor, NY 11724, USA

[2]Physiology Department and Donnelly Centre for Cellular and Biomolecular Research, University of Toronto, Toronto, ON, Canada

*Corresponding author: Jesse Gillis – jesse.gillis@utoronto.ca

Supplemental Figures

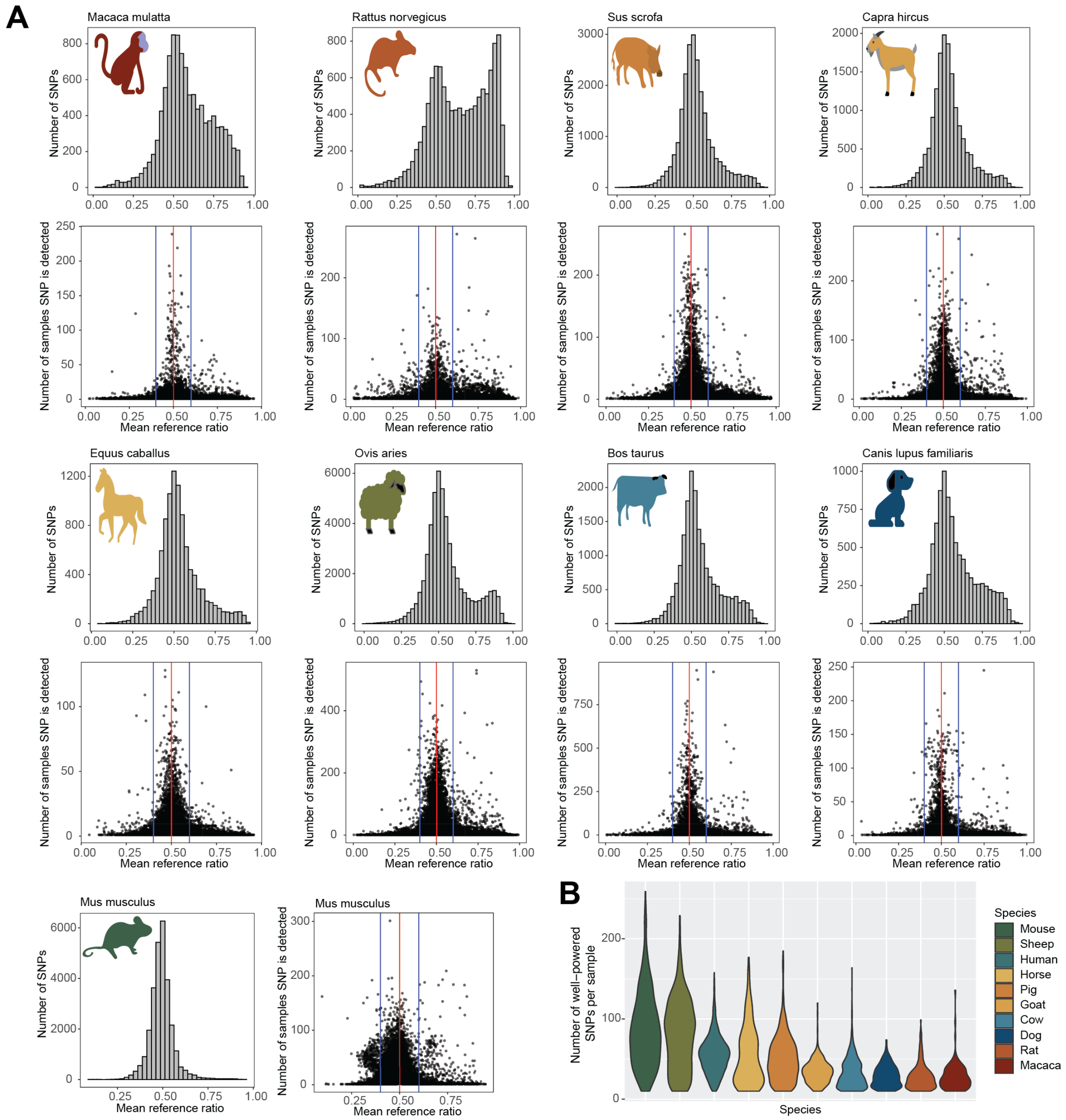

Supplemental Figure 1: Reference bias varies across individual SNPs

A Top histogram depicts the distribution of mean reference ratios for all detected SNPs in each species. The bottom scatter plot depicts the mean reference ratio against the sample size for each SNP. We exclude all SNPs from XCI ratio modeling whose mean reference ratio is < 0.40 or > 0.60 (blue lines), indicating consistent bias in allelic expression for either the alternate or reference allele.

B Violin plots depicting the distribution of the number of filtered SNPs (see methods) per sample for each species, where we require a minimum of 10 SNPs for XCI ratio modeling. Details for source data are provided in the Data and Code Availability statements.

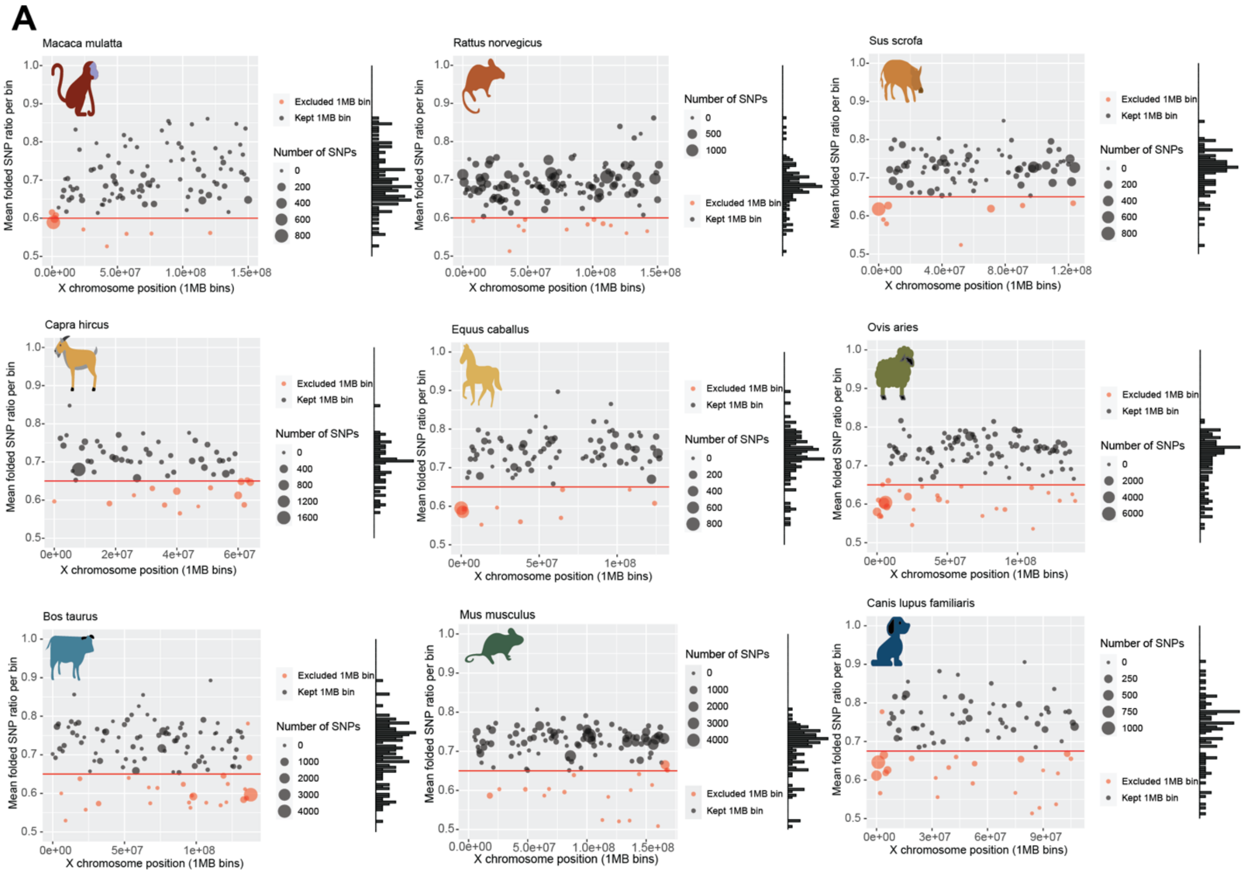

Supplemental Figure 2: Escape from XCI is enriched in chromosomal ends
A Scatter plots comparing the chromosomal location (1 mega-base bins) and the mean folded allelic expression ratio for all SNPs within each 1MB bin, derived from samples with skewed XCI (see methods). The marginal histogram depicts the distribution of mean folded allelic expression ratios per 1MB bin. The size of the data points corresponds to the number of SNPs in each 1MB bin. Red data points indicate 1MB bins that were excluded from analysis as probable escape regions, due to balanced allelic expression in samples with skewed XCI ratios. The chromosomal ends of all species, except rat, exhibit large clusters of SNPs with escape signal, likely pseudo-autosomal regions. The red lines depict the threshold of allelic-expression used to classify 1MB bins as escape or not. Details for source data are provided in the Data and Code Availability statements.

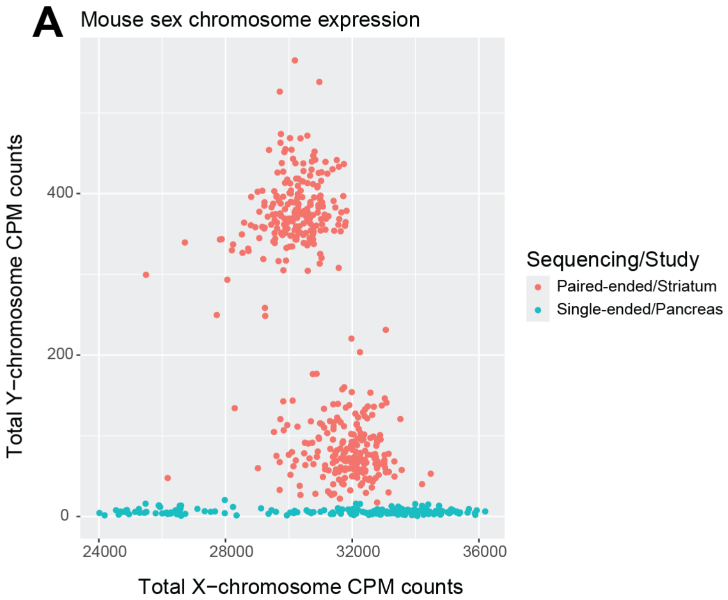

Supplemental Figure 3: Determining mouse sex sample annotations

A Scatter plot comparing the total number of Counts Per Million (CPM) counts of the X-chromosome (X-axis) and the Y-chromosome (Y-axis) for the two Diversity Outbred mice studies. The study that sampled the pancreas used single-ended sequencing and only sampled females, while the study that sampled the striatum used paired-ended sequencing and sampled a mix of males and females. Samples with <200 Y-chromosome CPM counts were labeled as female. Details for source data are provided in the Data and Code Availability statements.

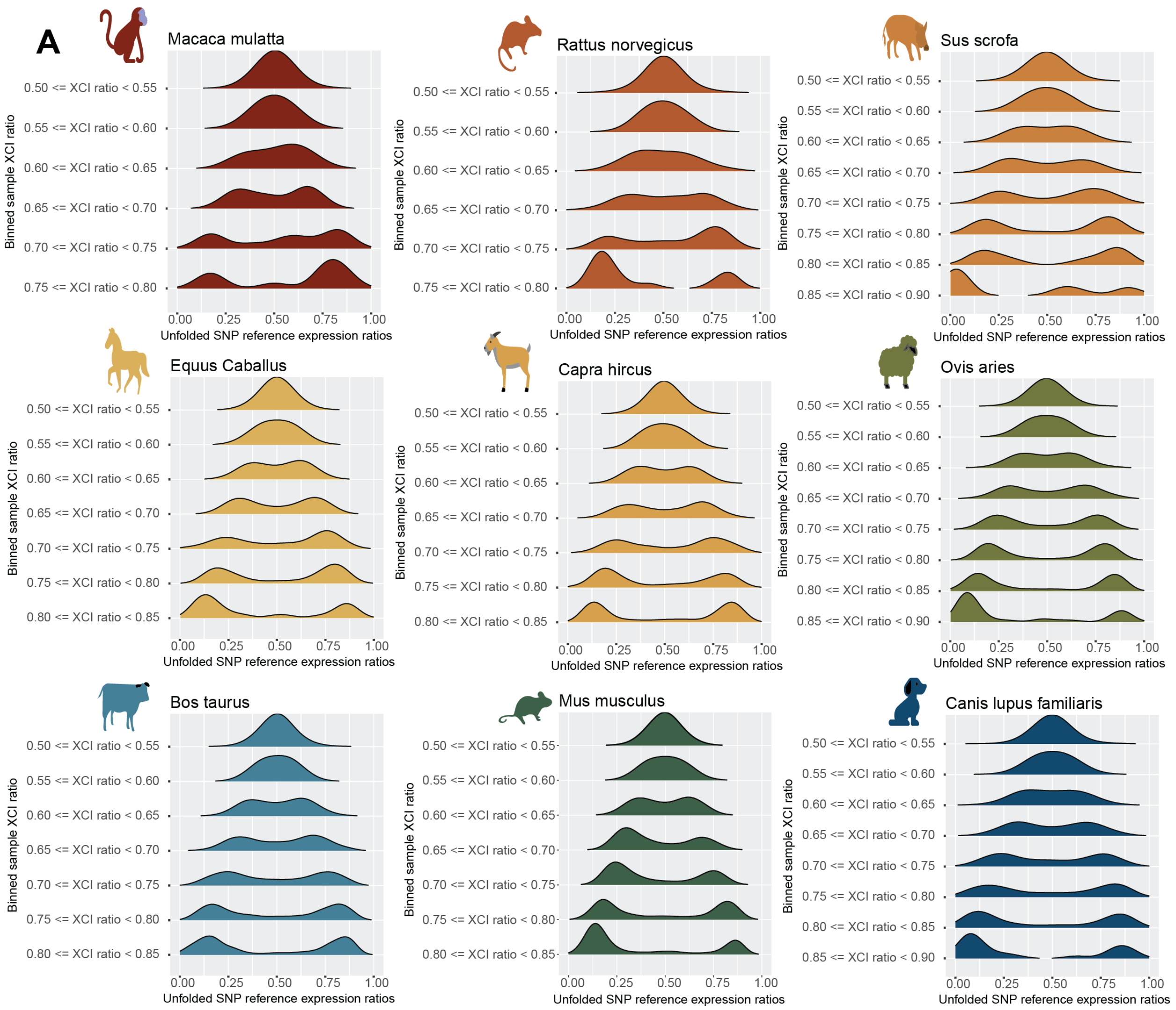

Supplemental Figure 4: Reference allelic expression distributions exhibit bi-parental haplotype expression signatures expected of the X-chromosome
A Density distributions of reference allelic expression ratios aggregated across samples binned by their estimated XCI ratio, ordered from balanced to more extreme XCI ratios (top to bottom). For samples with balanced XCI, the parental haplotypes cannot be distinguished, with clear separation of the parental haplotypes as the XCI ratio increases. Details for source data are provided in the Data and Code Availability statements.

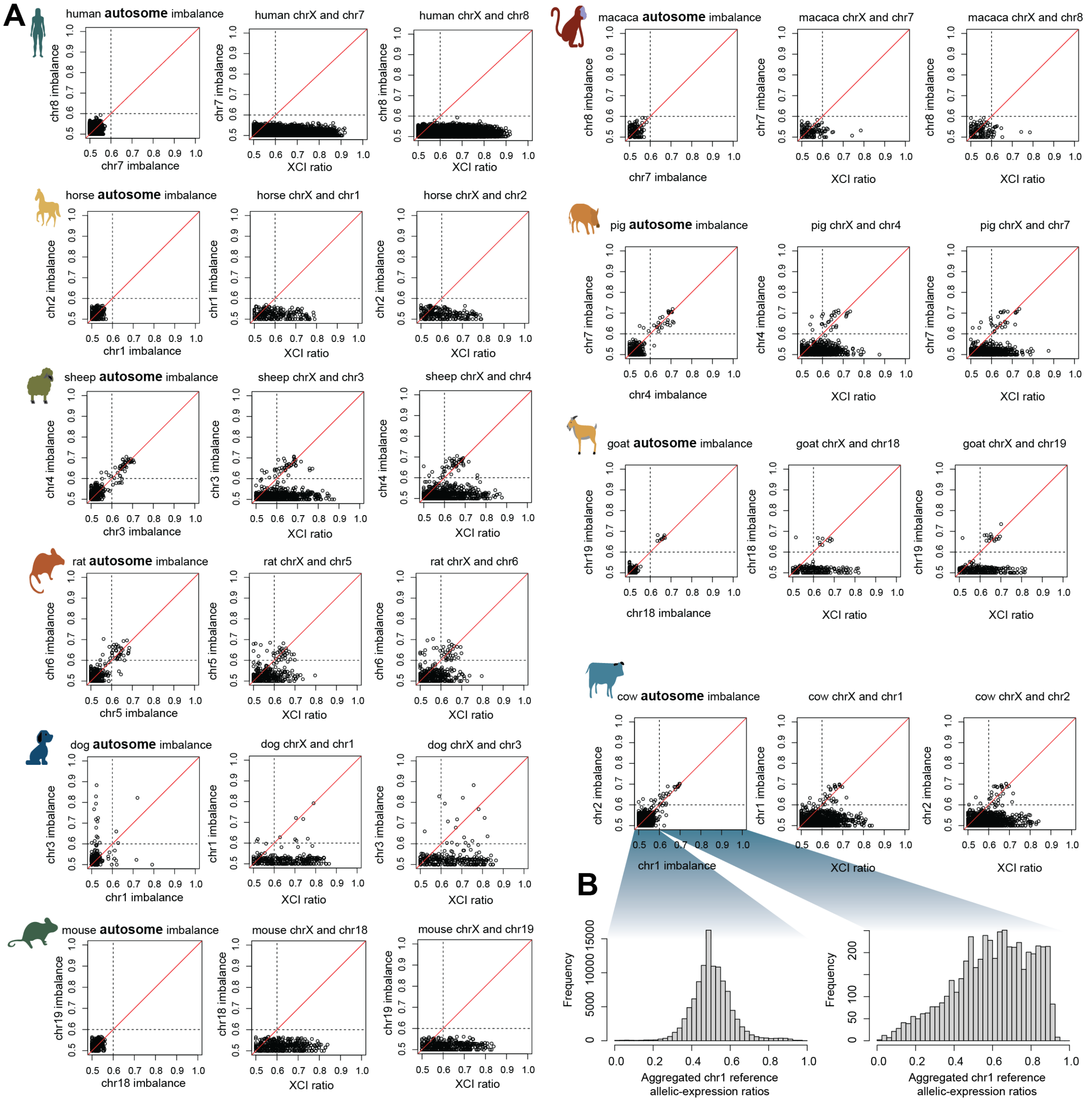

Supplemental Figure 5: Comparing autosomal and X chromosome allelic imbalances

A Each data point is a single individual sample. For each species, the first scatter plot compares the aggregated allelic imbalance of two autosomes (see methods). The following two scatter plots compare the aggregated allelic imbalance of an autosome and the XCI ratio for each sample. Samples that exhibited imbalanced allelic expression on an autosome were excluded from analysis, using a threshold of an imbalance >= 0.60 (dotted lines).

B Histogram of the reference allelic-expression ratios of all chromosome 1 SNPs from the Cow samples with a chromosome 1 autosomal imbalance either < 0.60 (left) or >= 0.60 (right). The large autosomal imbalances can be attributed to extensive reference bias in allele-specific expression ratios. Cow results are representative of all other species. Details for source data are provided in the Data and Code Availability statements.

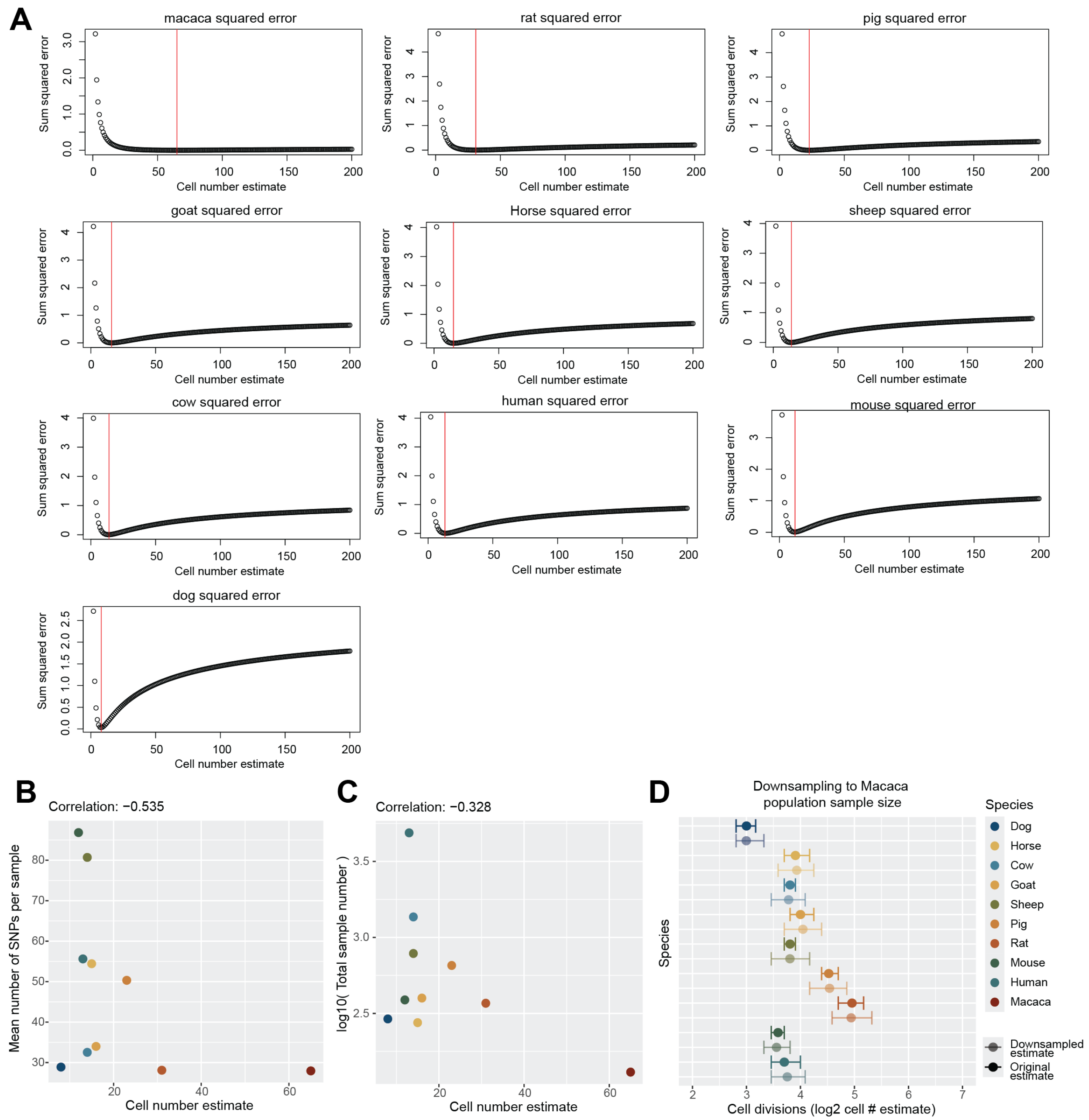

Supplemental Figure 6: Estimating embryonic cell counts from population XCI ratio variance

A Plots comparing the normal model associated with the estimated number of cells present during embryonic lineage specification (x-axis, see methods) to the sum of squared error between the percentiles of the tails of the empirical population XCI ratio distribution and the theoretical normal model (y-axis, see methods). The red lines depict the normal model with minimum error and the associated cell number estimate for each species.

B Scatter plot comparing the cell number estimate (x-axis) to the mean number of SNPs per sample (y-axis) for each species. The correlation (spearman) between the two is -0.535.

C Scatter plot comparing the cell number estimate (x-axis) to the total number of samples (y-axis, log10 scale) for each species. The correlation (spearman) between the two is -0.328.
D Plot comparing cell number estimates for each species, excluding macaca, for original (bold) and down sampled (transparent) data. Species sample sizes were down sampled to 130 to match the macaca sample size (see methods). Error bars are 95% confidence intervals computed through bootstrap simulations with n = 2000, with the measure of center corresponding to the estimated cell number per species on a log-2 scale. Details for source data are provided in the Data and Code Availability statements.

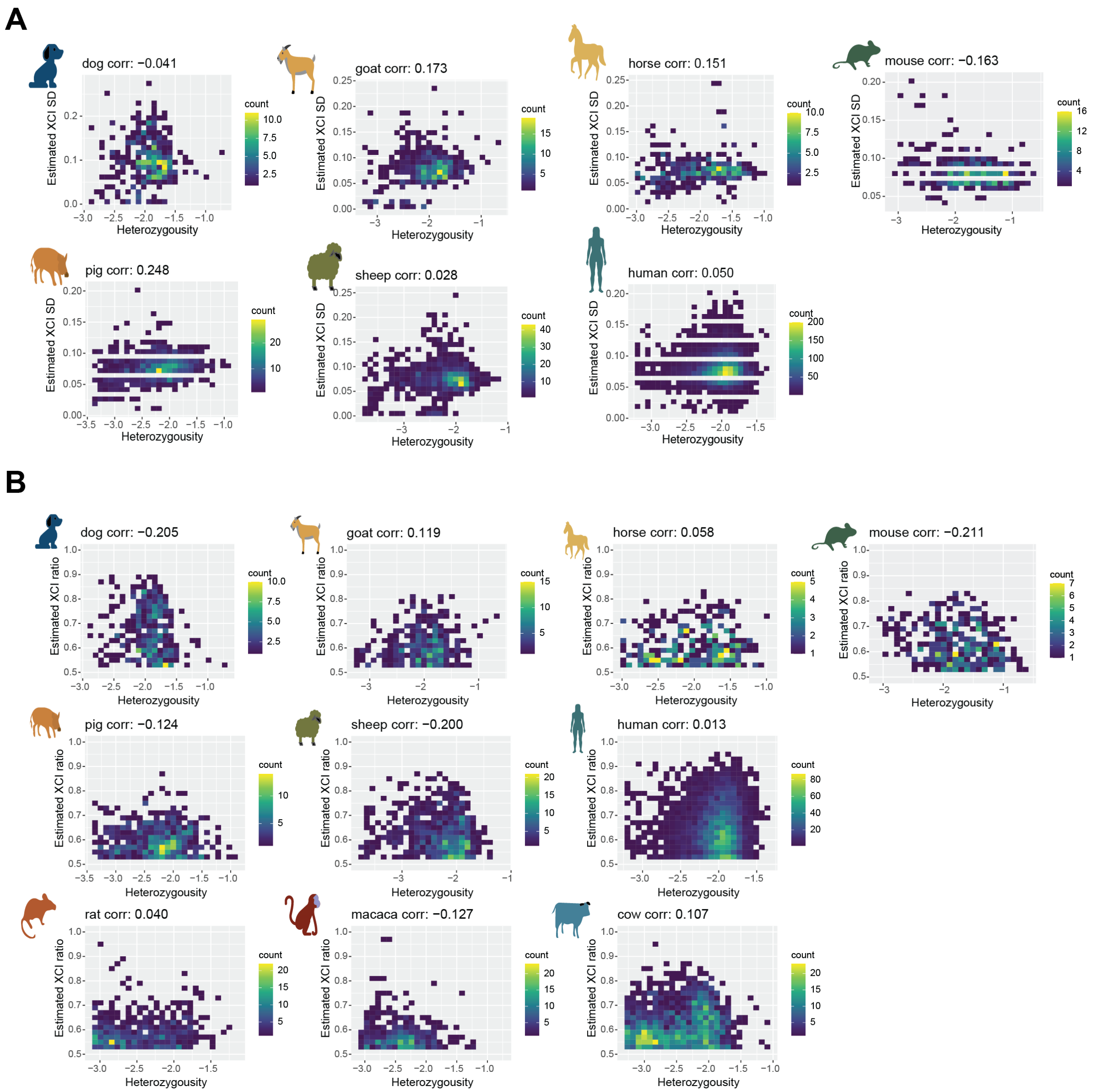

Supplemental Figure 7: Species with no association between sample heterozygosity and variance in X-linked allelic expression

A Binned scatter plots comparing the sample heterozygosity (log-10 of the number of SNPs per sample divided by the number of unique SNPs detected per species) to the estimated standard deviation (SD) in X-linked allelic expression (SD of the maximum-likelihood folded-normal distribution per sample). Spearman correlation coefficients are presented next to the species' names. Color bars represent the number of datapoints per 2D bin.

B Binned scatter plots comparing the sample heterozygosity (log-10 of the number of SNPs per sample divided by the number of unique SNPs detected per species) to the estimated XCI ratio (mean of the maximum-likelihood folded-normal distribution per sample). Spearman correlation coefficients are presented next to the species' names.

Color bars represent the number of datapoints per 2D bin. Source data are provided as a Source Data file.

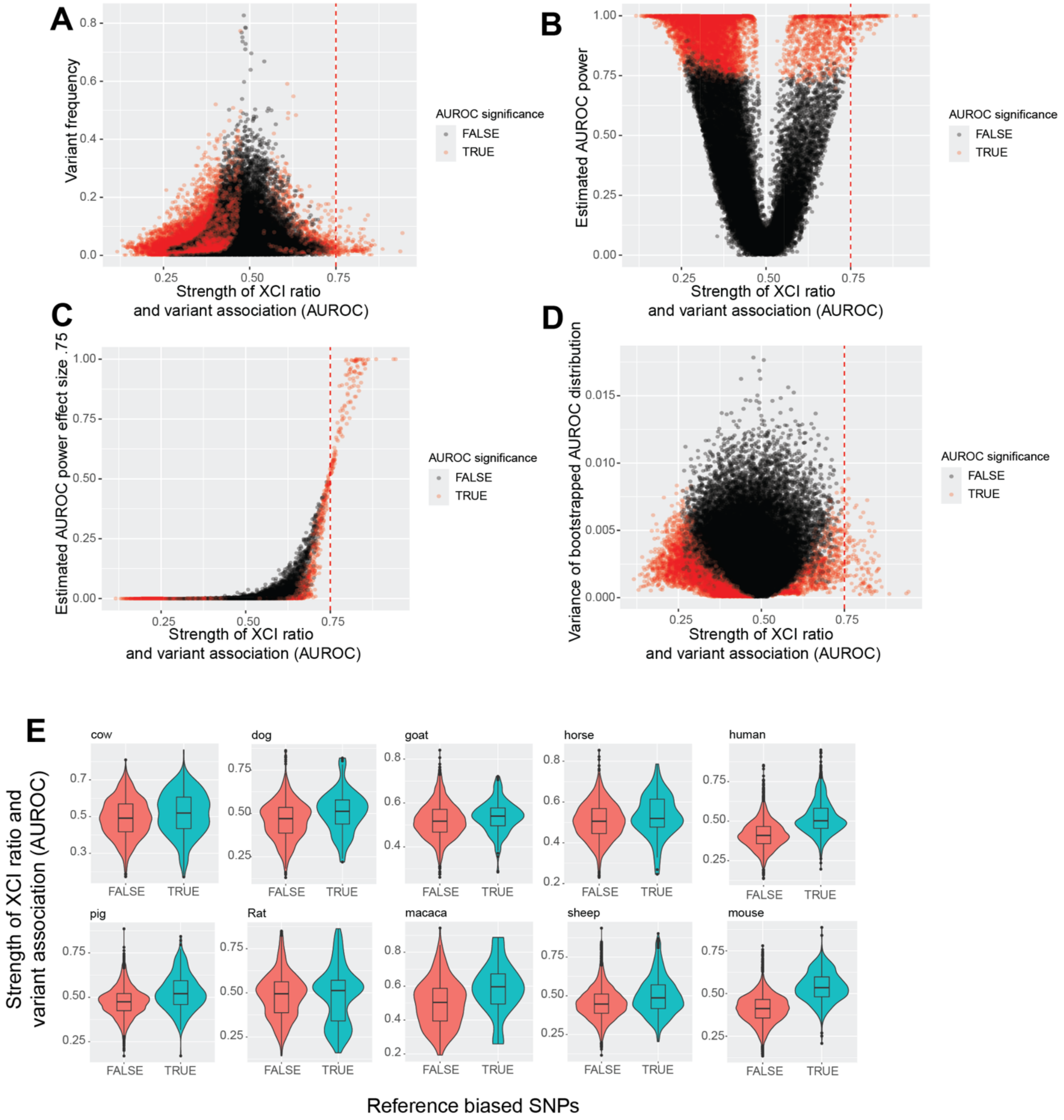

Supplemental Figure 8: Low frequency variants are powered to detect significant associations with XCI ratios

A Scatter plot comparing the initial AUROC against variant frequency for all variants across all species. Statistical significance of AUROCs is determined by an FDR-corrected p-value <= 0.05. The red dotted line in all 4 figure panels represents the AUROC threshold used to determine individual variants with a moderate association with XCI ratios.

B Scatter plot comparing the initial AUROC against the estimated power to detect a significant effect for each variant. Power was estimated through bootstrap simulations using a significance threshold of p-value <= 0.05, see methods.
C Scatter plot comparing the initial AUROC against the estimated power to detect an AUROC with effect size 0.75 or greater. Power was estimated through bootstrap simulations, see methods.
D Scatter plot comparing the initial AUROC against the variance of the bootstrapped distribution of AUROCs for each variant, see methods.
E Violin and boxplots depicting the distributions of AUROCs for the SNPs classified as either reference biased or not from the analysis in Supp. Fig. 1. Box plots indicate median (middle line), 25th, 75th percentile (box) and 1.5 times the inter-quartile range from the first and third quartiles (whiskers) with outliers as single points. Details for source data are provided in the Data and Code Availability statements.

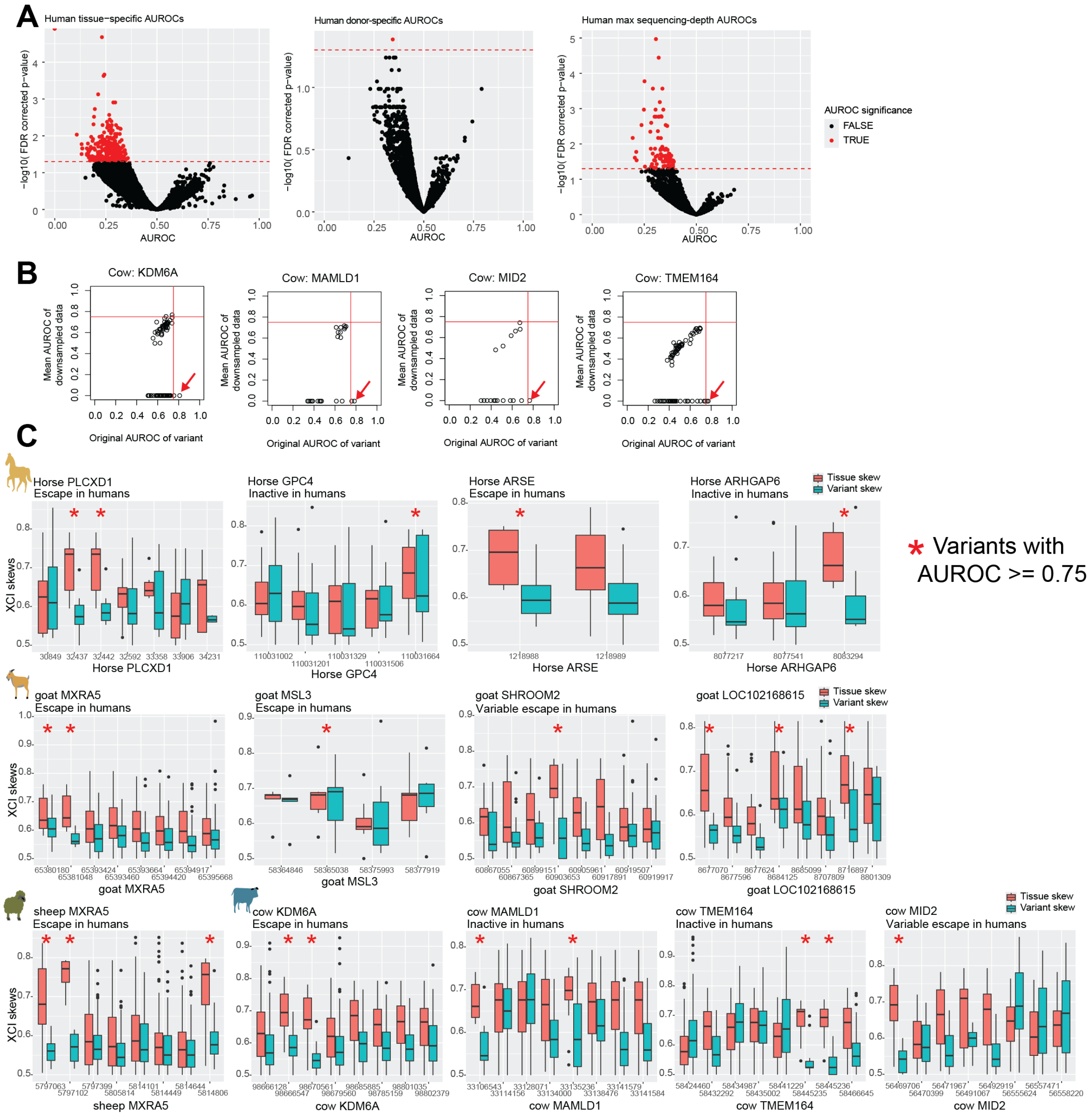

Supplemental Figure 9: Human variant XCI ratio associations and example cross-variant allelic expression ratios within individual genes

A Scatter plots comparing the AUROC (x-axis) of a variant to its FDR-corrected p-value (y-axis) for human variants only. P-values are reported in negative log-10 scale. Variants with an FDR-corrected p-value <= 0.05 are highlighted in red. The plots report the statistics for human variants assessed in a tissue-specific manner (leftmost), donor-specific manner (center), and when using the sample per donor with the highest sequencing depth (rightmost).

B Scatter plots comparing the original AUROC (x-axis) to the mean AUROC using down sampled data (y-axis) for variants within the 4 cow genes that reported significant XCI

ratio associations (AUROC >= 0.75, red arrows). Red lines indicate the 0.75 threshold, all of the variants with original AUROCs >= 0.75 are not detected in the down sampled data.
C Boxplots comparing the estimated XCI ratios of tissues (red, y-axis) and the allelic-ratios of individual variants (blue, y-axis) within those tissues across all variants detected within a given gene (x-axis). We provide examples of genes for several species that contain at least 1 variant we identify as significantly associated with XCI ratios (AUROC >= 0.75, red asterisks correspond to an FDR-corrected p-value <= 0.05). Escape annotations are from Supp. Table 1 here[49]. Box plots indicate median (middle line), 25th, 75th percentile (box) and 1.5 times the inter-quartile range from the first and third quartiles (whiskers) with outliers as single points. Details for source data are provided in the Data and Code Availability statements.

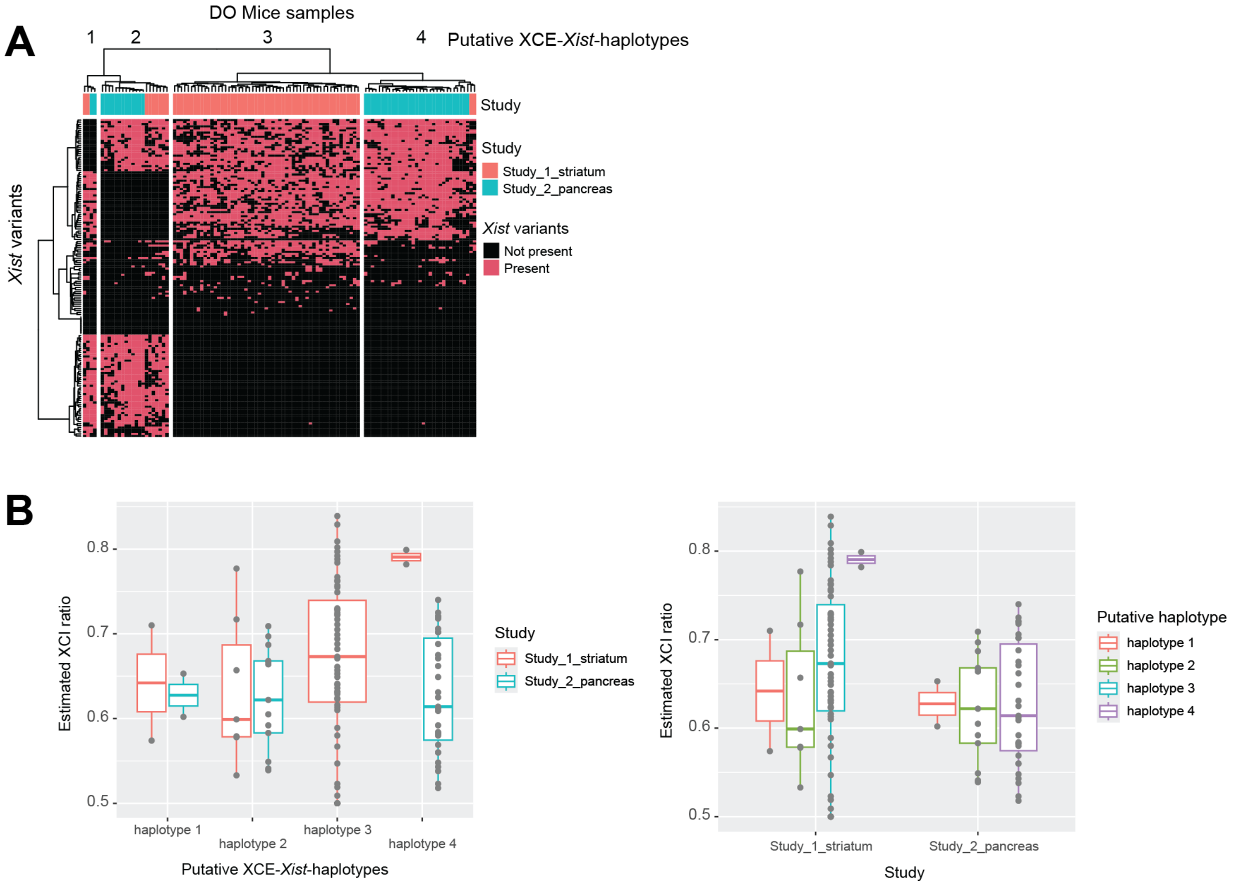

Supplemental Figure 10: Putative mouse XCE-*Xist*-haplotypes exhibit highly variable XCI ratios

A Hierarchically clustered heatmap of *Xist* variants (rows) and Diversity Outbred mice samples (columns). Red indicates that *Xist* variant is present within the given sample, where black indicates that *Xist* variant is not detected. Sample study annotations are provided as the column color bar. The column dendrogram is cut into 4 groups to capture the clear sample groupings present.

B Boxplots comparing the estimated XCI ratios (y-axis) of DO mice samples grouped by their putative XCE-Xist-haplotypes and study/tissue (x-axes). Box plots indicate median (middle line), 25th, 75th percentile (box) and 1.5 times the inter-quartile range from the first and third quartiles (whiskers) of the data points (grey points). Details for source data are provided in the Data and Code Availability statements.
