# Supplementary material for: Population variability in X-chromosome inactivation across 10 mammalian species
Source: Nat Commun. 2024 Oct 18;15:8991. doi: 10.1038/s41467-024-53449-1 (PMC11487087; doi:10.1038/s41467-024-53449-1)
Supplement: Supplementary file 3 — Reporting Summary [file 41467_2024_53449_MOESM3_ESM.pdf]

nature portfolio

Corresponding author(s): Jesse Gillis

Last updated by author(s): Sep 17, 2024

Reporting Summary

Nature Portfolio wishes to improve the reproducibility of the work that we publish. This form provides structure for consistency and transparency in reporting. For further information on Nature Portfolio policies, see our Editorial Policies and the Editorial Policy Checklist.

Statistics

For all statistical analyses, confirm that the following items are present in the figure legend, table legend, main text, or Methods section.

n/a	Confirmed	
☐	☒	The exact sample size (*n*) for each experimental group/condition, given as a discrete number and unit of measurement
☐	☒	A statement on whether measurements were taken from distinct samples or whether the same sample was measured repeatedly
☐	☒	The statistical test(s) used AND whether they are one- or two-sided<br>*Only common tests should be described solely by name; describe more complex techniques in the Methods section.*
☒	☐	A description of all covariates tested
☐	☒	A description of any assumptions or corrections, such as tests of normality and adjustment for multiple comparisons
☐	☒	A full description of the statistical parameters including central tendency (e.g. means) or other basic estimates (e.g. regression coefficient) AND variation (e.g. standard deviation) or associated estimates of uncertainty (e.g. confidence intervals)
☐	☒	For null hypothesis testing, the test statistic (e.g. *F*, *t*, *r*) with confidence intervals, effect sizes, degrees of freedom and *P* value noted<br>*Give P values as exact values whenever suitable.*
☒	☐	For Bayesian analysis, information on the choice of priors and Markov chain Monte Carlo settings
☒	☐	For hierarchical and complex designs, identification of the appropriate level for tests and full reporting of outcomes
☐	☒	Estimates of effect sizes (e.g. Cohen's *d*, Pearson's *r*), indicating how they were calculated

Our web collection on statistics for biologists contains articles on many of the points above.

Software and code

Policy information about availability of computer code

Data collection	All non-human mammalian fastq data was downloaded from the Sequencing Read Archive (SRA, https://www.ncbi.nlm.nih.gov/sra), where only samples annotated as female were selected, using the metadata provided through SRA. The entire sample processing pipeline uses a standard collection of bioinformatics software tools, all available for installation via Conda (STAR v2.7.9a, GATK v4.2.2.0, samtools v1.13, igvtools v2.5.3, and sra-tools 2.11.0). All Snakemake workflow rules, environment setup procedure, analysis commands and options, and underlying libraries are available on Github at https://github.com/gillislab/cross_mammal_xci , and https://github.com/gillislab/xskew.
Data analysis	All analysis was performed in R v4.3.3. All plots were generated using ggplot269 v3.4.2 functions. All associated code can be found at https://github.com/gillislab/cross_mammal_xci/tree/main/R. Code for generating all figure panels using associated source data can be found at https://github.com/gillislab/cross_mammal_xci/blob/main/R/figure_plots_with_data_code.md. The snakemake pipeline used for processing the non-human mammalian data can be found at https://github.com/gillislab/cross_mammal_xci/tree/main. DOI: 10.5281/zenodo.13774726. Details and code for processing the human GTEx samples can be found here (reference: 28).

For manuscripts utilizing custom algorithms or software that are central to the research but not yet described in published literature, software must be made available to editors and reviewers. We strongly encourage code deposition in a community repository (e.g. GitHub). See the Nature Portfolio guidelines for submitting code & software for further information.

Data

Policy information about availability of data

All manuscripts must include a data availability statement. This statement should provide the following information, where applicable:

- Accession codes, unique identifiers, or web links for publicly available datasets
- A description of any restrictions on data availability
- For clinical datasets or third party data, please ensure that the statement adheres to our policy

The source data for all figure panels can be found at https://github.com/gillislab/cross_mammal_xci/tree/main/R/data_for_plots. Where applicable, exact p-values are provided in the source data files. The SRA accession numbers for all non-human mammalian samples processed can be found at https://github.com/gillislab/cross_mammal_xci/blob/main/R/data_for_plots/all_keep_species_meta.Rdata. Details for accessing the GTEx samples can be found here https://gtexportal.org/home/protectedDataAccess.

Research involving human participants, their data, or biological material

Policy information about studies with human participants or human data. See also policy information about sex, gender (identity/presentation), and sexual orientation and race, ethnicity and racism.

Reporting on sex and gender	All human data utilized was obtained from the Genotype Tissue Expression (GTEx) dataset. All reporting on sex and gender can be found from the original study. Only female samples were used in this work.
Reporting on race, ethnicity, or other socially relevant groupings	All human data utilized was obtained from the Genotype Tissue Expression (GTEx) dataset. All reporting on race and ethnicity can be found from the original study.
Population characteristics	All human data utilized was obtained from the Genotype Tissue Expression (GTEx) dataset. All reporting on population characteristics can be found from the original study.
Recruitment	All human data utilized was obtained from the Genotype Tissue Expression (GTEx) dataset. All reporting on recruitment can be found from the original study.
Ethics oversight	All human data utilized was obtained from the Genotype Tissue Expression (GTEx) dataset. All ethics information can be found from the original study.

Note that full information on the approval of the study protocol must also be provided in the manuscript.

Field-specific reporting

Please select the one below that is the best fit for your research. If you are not sure, read the appropriate sections before making your selection.

[x] Life sciences   [ ] Behavioural & social sciences   [ ] Ecological, evolutionary & environmental sciences

For a reference copy of the document with all sections, see nature.com/documents/nr-reporting-summary-flat.pdf

Life sciences study design

All studies must disclose on these points even when the disclosure is negative.

Sample size	All data was downloaded from the Sequencing Read Archive. Sample sizes were established through successful download and processing of data, sample sizes were not determined empirically. We processed all available female annotated samples on SRA for the non-human mammalian species, except for mouse. Mouse sample size was determined by the number of samples made available from the two Diversity Outbred mouse studies we sourced data from (references 42 and 43 in manuscript), which supplied a final mouse sample size comparable to the other non-human mammalian species. We processed all available female human samples from GTEx.
Data exclusions	Data were excluded on the basis of quality control filters, namely requiring at least 10 well-powered heterozygous SNPs and no evidence for global allelic imbalances as determined by chromosome-wide assessments of the X-chromosome and 2 autosomes per sample.
Replication	This work reports population-scale assessments of X-chromosome variability for several mammalian species, of which comparable independent population-scale data for assessments of replicability are limited.
Randomization	Randomization was not relevant for this study
Blinding	Blinding was not relevant to this study

Reporting for specific materials, systems and methods

We require information from authors about some types of materials, experimental systems and methods used in many studies. Here, indicate whether each material, system or method listed is relevant to your study. If you are not sure if a list item applies to your research, read the appropriate section before selecting a response.

Materials & experimental systems

n/a	Involved in the study
☒	☐ Antibodies
☒	☐ Eukaryotic cell lines
☒	☐ Palaeontology and archaeology
☐	☒ Animals and other organisms
☒	☐ Clinical data
☒	☐ Dual use research of concern
☒	☐ Plants

Methods

n/a	Involved in the study
☒	☐ ChIP-seq
☒	☐ Flow cytometry
☒	☐ MRI-based neuroimaging

Animals and other research organisms

Policy information about studies involving animals; ARRIVE guidelines recommended for reporting animal research, and Sex and Gender in Research

Laboratory animals	All raw data was downloaded from the Sequencing Read Archive. Samples were not filtered by strain, only by species. The metadata provided by SRA was used for identifying relevant samples.
Wild animals	*Provide details on animals observed in or captured in the field; report species and age where possible. Describe how animals were caught and transported and what happened to captive animals after the study (if killed, explain why and describe method; if released, say where and when) OR state that the study did not involve wild animals.*
Reporting on sex	All raw data was downloaded from the Sequencing Read Archive. Only samples annotated as female from the metadata provided from SRA were included in this study.
Field-collected samples	*For laboratory work with field-collected samples, describe all relevant parameters such as housing, maintenance, temperature, photoperiod and end-of-experiment protocol OR state that the study did not involve samples collected from the field.*
Ethics oversight	All raw data was downloaded from the Sequencing Read Archive, all required ethics oversights can be found in the original studies.

Note that full information on the approval of the study protocol must also be provided in the manuscript.

Plants

Seed stocks	*Report on the source of all seed stocks or other plant material used. If applicable, state the seed stock centre and catalogue number. If plant specimens were collected from the field, describe the collection location, date and sampling procedures.*
Novel plant genotypes	*Describe the methods by which all novel plant genotypes were produced. This includes those generated by transgenic approaches, gene editing, chemical/radiation-based mutagenesis and hybridization. For transgenic lines, describe the transformation method, the number of independent lines analyzed and the generation upon which experiments were performed. For gene-edited lines, describe the editor used, the endogenous sequence targeted for editing, the targeting guide RNA sequence (if applicable) and how the editor was applied.*
Authentication	*Describe any authentication procedures for each seed stock used or novel genotype generated. Describe any experiments used to assess the effect of a mutation and, where applicable, how potential secondary effects (e.g. second site T-DNA insertions, mosiacism, off-target gene editing) were examined.*
